# Cultural Differences in Mixed Emotions: The Role of Dialectical Thinking

**DOI:** 10.3389/fpsyg.2020.538793

**Published:** 2021-01-11

**Authors:** Wen Zheng, Ailin Yu, Disi Li, Ping Fang, Kaiping Peng

**Affiliations:** ^1^Department of Medical Psychology, Capital Medical University, Beijing, China; ^2^Department of Psychology, Tsinghua University, Beijing, China; ^3^The Professional Qualification Authority of Ministry of Transport, Beijing, China; ^4^College of Psychology, Capital Normal University, Beijing, China

**Keywords:** culture, dialectical thinking, mixed emotions, discomfort, thinking style

## Abstract

Who can feel both happy and sad at the same time, but not discomfort? This study aimed to investigate the cultural differences in mixed emotional experiences induced by conflict stimuli among American and Chinese undergraduate students. In total, 160 Americans and 158 Chinese watched two different valence advertisements (one predominantly positive and the other predominantly negative) that elicited mixed emotions; their feelings were assessed through self-reported measures. Findings indicated the impact that cultural differences have in people’s mixed emotional experiences depends on the emotional components of the mixed emotional situations. The Americans and Chinese both experience a comparably intense mixture of emotions in different valence situations, but their discomfort toward conflicting stimuli is different. Further, dialectical thinking may be a mechanism behind the influence of cultural differences in people’s mixed emotional experiences. Implications for emotion theory and research are discussed.

## Introduction

On November 16, 2015, Oxford Dictionaries announced that their “Word of the Year” was an emoji: “Face with Tears of Joy.” It was the first time that Oxford Dictionaries chose a pictograph as its “Word of the Year,” representing a break with past winners, which were mostly old-fashioned, string-of-letters-type words. This emoji represents the experience of sadness mixed with joy, a “bittersweet” feeling. *Mixed emotions* represent a subset of emotion blends, which were defined as the co-occurrence of any two or more *same-valence* or *opposite-valence emotions* (Larsen and McGraw, 2011, 2014). This study surveyed 160 American and 157 Chinese undergraduate students as well as private sector employees to examine cultural differences in the experience of mixed emotions between the two groups. They were assessed through self-reported measures, using different valence advertisements as means to reflect peoples’ complex emotional experiences in response to emotion inducing stimuli.

Previous research demonstrates that the experience of mixed emotions is influenced by response format (Schimmack et al., 2001, unpublished), one’s *openness* (de Rivera, 1984; Tang and Singer, 1997), resilience (Ong and Bergeman, 2004), and age (Coats and Blanchard-Fields, 2008; Ersner-Hershfield et al., 2008). Moreover, mixed emotions have a positive impact on a person’s well-being; hence, it may buffer the negative consequences of stressful events on an individuals’ health (Berrios et al., 2018). Some researchers began to focus on the influence of *culture* on the experience of mixed emotions, suggesting that *East Asians* experience more mixed emotions than *North Americans* (Bagozzi et al., 1999; Kitayama et al., 2000). Thus, our study targets this research question. We aimed to investigate *cultural differences* in the *experience of mixed emotions*, as well as to explain how the *dialectical thinking style* may affect undergraduate students’ experiences of mixed emotions.

Cross-cultural studies have repeatedly documented that the *Western* and the *Eastern epistemologies* and *systems of thought* diverge in important ways (Peng and Nisbett, 1999; Hamamura et al., 2008; Miyamoto et al., 2010). *Eastern philosophies* are largely rooted in *Confucianism*, *Taoism*, and *Buddhism*, which can be described as *dialectical*. Dialectical thinking features the three principles of *contradiction* (i.e., two opposing positions can both be true), *change* (i.e., two opposing positions may lie on different points of a temporal continuum), and *holism* (i.e., all things in the universe are interrelated). *Western culture*, which is largely based on *Greek* and *Aristotelian logic*, can be characterized as *linear* or *synthesis-oriented*. These logical thinking styles feature three key principles: the *law of identity* (i.e., if anything is true, then it is true), the *law of excluded middle* (i.e., any statement is either true or false), and the *law of non-contradiction* (i.e., no statement can be both true and false; Peng and Nisbett, 1999). This thinking tradition tends to limit the degree to which Westerners engage in dialectical processing.

A growing number of cross-cultural research studies indicate that East Asians engage in dialectical thinking, and that this propensity may also influence their emotions (Spencer-Rodgers et al., 2010). People in *independent-based cultures* (e.g., the United States) tend to experience emotions in *opposite ways*, whereas people in *interdependent-based cultures* (e.g., China) experience emotions in *dialectic ways* (Bagozzi et al., 1999). In summary, dialecticism sees opposite-valence emotions (e.g., mixed emotions) as compatible with each other, whereas Western philosophies consider these emotions to be *conflicting*.

Previous research mainly focused on two aspects of cultural differences in mixed emotions: the *frequency* and the *intensity* of the *mixed emotional experience*; Bagozzi et al. (1999) had Asian and American subjects recall and indicate the *frequency* with which they experienced positive and negative emotions and found that the frequency estimates of positive and negative emotions were positively correlated for Asians, whereas negative correlations were found for Americans. Kitayama et al. (2000) asked participants from Japan and America to estimate the *frequency* in which they experienced positive and negative emotions; they found similar correlations between positive and negative emotions: they were less negative in dialectic cultures than in non-dialectic cultures, and, based on the correlational indices of co-occurrence. This led to the conclusion that Asians experience mixed emotions more frequently than Westerners. However, although positive correlations between two variables may imply this, they co-occur frequently. For example, strong negative correlations do not necessarily imply that they co-occur less frequently (Zelenski and Larsen, 2000; Larsen et al., 2017). Additionally, there is a concern about what has been reported as “mixed emotions,” as specific reports might reflect people’s semantic knowledge about the stimulus they experience during scientific experiments (Russell, 2017; Itkes et al., 2019). Therefore, these aforementioned mixed emotion reports could have reflected people’s semantic judgments instead of their actual mixed emotional feelings.

Some researchers focused on how thinking style affects the *intensity* of the mixed emotional experience. To investigate the mechanism behind the notion that dialectical thinkers experience greater mixed emotions than non-dialectical thinkers, Spencer-Rodgers et al. (2010) experimented with Chinese and Euro-American undergraduates by making them experience mixed emotion life events. The results showed that Chinese people tend to engage in dialectical thinking; they exhibit greater mixed emotional experience than Euro-Americans, and dialecticism mediated participants’ mixed emotional experiences, reinforcing the significance of cultural differences. However, Leu et al. (2010) found no evidence that dialectical thinkers experience more mixed emotions than non-dialectical thinkers. In their study, participants reported their emotional response toward standardized positive, negative, and mixed situations that depicted episodes in a protagonist’s daily life. Results showed that cultural differences in the opposing emotions associations were found in positive events, and not in mixed or negative ones.

We may infer from the inconsistency of the aforementioned studies that the overall valence of the situation may have a mediation effect on how cultural differences affect mixed emotional experiences. Indeed, Miyamoto et al. (2010) found that the Japanese reported more mixed emotions than the Americans in *predominantly pleasant situations* (PPS), whereas there were no cultural differences in mixed emotions in *predominantly unpleasant situations* (*PUS*) or the mixed situations. Corroborating Miyamoto’s findings, Hui et al. (2009) had Chinese participants report their emotional experiences to a positive and a negative event every week over 15 weeks, and they found an interaction between dialectical thinking and event valence. Specifically, non-dialectical thinkers tended to experience more mixed emotions in positive than in negative events.

Additionally, traditional model on mixed emotional experience concentrated on the associated negative consequences (e.g., anxiety and stress; Williams and Aaker, 2002). According to the Cognitive Dissonance Theory (Festinger, 1957), the experience of conflict creates uncomfortable tensions or discomfort; Chinese, who are more prone to dialectical thinking, are more likely to accept contradiction in reality and to synthesize contradiction than Westerners, who are more prone to linear thinking (Peng and Nisbett, 1999). Thus, cultural differences in the attitude toward contradictions manifest themselves through emotional constructs, which are manifested based on the extent to which the contradictory elements elicit them; for example, Westerners exhibit tension or discomfort when presented with situations that elicit cognitive, emotional, or behavioral contradictions (Cacioppo and Berntson, 1994). In that topic, Oceja and Carrera (2009) investigated different processing patterns of mixed emotions and found that highly simultaneous pattern elicited greater tension than sequential and prevalent patterns.

In order to account for the emotional nature of a given situation (Miyamoto et al., 2017), we induced mixed emotions by presenting two different advertisements with varying valences (Williams and Aaker, 2002). We hypothesized that (H1) both the Chinese and American people are capable of experiencing mixed emotions, but the intensity of the experience varies depending on the situation; specifically, in PPS, the Chinese experienced more mixed emotion than the Americans, whereas in PUS, the Americans experienced more mixed emotion than the Chinese. Notably, the Chinese are more inclined to engage in dialectical thinking, and they tend to deal with apparent contradictions in a compromising way, which may advocate tolerance of more blended emotions. Thus, we inferred that there is a difference between how the Americans and Chinese experience contradictions. Further, we have also tried to explain how the dialectical thinking style may affect the discomfort elicited by confronting opposing emotional stimuli. We hypothesized that (H2) the Chinese will tend to engage in dialectical thinking, and in PPS, it will be easier for them to identify negative meanings in positive events; as such, they will experience more mixed emotions and discomfort. Contrastingly, Americans will tend to engage in linear thinking, and in PPS, they will try to amplify the significance of positive emotions, thus reducing the impact of negative emotions; in doing so, they will experience less intense mixed emotions regarding the same situation. However, in PUS, both the Chinese and Americans may experience the same level of mixed emotion and discomfort. Owing to this, the Chinese try to find positive meanings in negative events, whereas the Americans have to be motivated to regulate their negative emotions. Regarding mixed emotion calculation, early studies used variable correlations to index mixed emotional experience (Russell, 1980; Watson and Tellegen, 1985; Remmington et al., 2000). To address the limitations of using correlations, we applied two co-current indexes to indicate the intensity of mixed emotional experience.

## Materials and Methods

### Participants

In total, 160 American individuals participated in this study. Among them, there were 57 undergraduates (18 males, 39 females, mean age = 20.32 years) from the University of California, Berkeley, and 103 individuals (55 males, 48 females, mean age = 39.35 years) were recruited from Amazon Mechanical Turk. All the American participants were Anglo-, African-, Hispanic-, or Indian-Americans, including 80.0% Anglo-Americans, 11.25% African-Americans, 6.25% Hispanic-Americans, and 2.5% Indian-Americans. In total, 158 Chinese individuals participated in this study. Among them, there were 122 undergraduate students (32 males, 90 females, mean age = 19.80 years) from the Capital Normal University in Beijing, China, and 36 individuals (20 males, 16 females, mean age = 29.74 years) were recruited from an advanced technological enterprise in Shanghai. Undergraduate participants were recruited from both universities’ Introduction to Psychology for course credit. American participants from Amazon Mechanical Turk and Chinese workers were paid.

This study was carried out in accordance with the recommendations of the guidelines of Human Research Ethics Committees of both the Capital Normal University and the University of California, Berkeley. All participants provided written informed consent prior to their participation. We affirm that such consent was in accordance with the Declaration of Helsinki. The protocol was approved by the Human Research Ethics Committees of both the Capital Normal University and the University of California Berkeley.

### Materials

#### Advertisements

Two advertisements were used in the study as *conflict stimuli* (Williams and Aaker, 2002). Each advertisement included a colorful photograph and a corresponding, contradictory advertising message (that reflected the opposite emotion of the image); this was intended to elicit two different emotional ambivalence states: predominantly pleasant and predominantly unpleasant mixed emotions, respectively.

Participants in the PPS read the message “My grandpa passed away years ago. He was a college professor and he dedicated his whole life to education. I loved spending time with him. I’m happy that he was alive long enough to get to know me and help raise me”; in the PUS, they read “I have been dreading this moment, but it has finally arrived. A chapter in my life is ending, and the future is still uncertain. I’ll miss the neighborhood and the friends I’ve made. I really do not want to leave. It’s a sad and nostalgic time.” This message elicits a predominantly unpleasant mixed emotion with stronger feelings of sadness than happiness.

To enhance external validity, positive-valence product content was included in the advertisement to enhance external validity and was consistently presented in all conditions. For example, one positive-valence content stated, “Watson color film has top color quality-plus; the texture will always be sharp, never grainy. Just like life.”

#### Dialectical Self Scale

The 32-item *Dialectical Self Scale* (DSS; Spencer-Rodgers et al., 2001, unpublished) was designed to assess subjects’ ability to think dialectically. The scale is composed of three subscales: contradiction, cognition change, and behavior change. Here is a sample item: “When I hear two sides of an argument, I often agree with both.” Participants rated each item on a 7-point Likert scale ranging from 1 (strongly disagree) to 7 (strongly agree). The DSS possesses adequate cross-cultural validity and reliability (Hamamura et al., 2008). In our study, the Cronbach’s *α* values were 0.69 (Chinese) and 0.87 (Americans).

#### Emotion Measurement

Participants were asked to imagine themselves as in the situations described in the advertisements. Then, they rated nine different emotions on a 7-point scale from 1 (not at all) to 7 (very strongly), such as “If you were the grandson, how sad would you feel?” Positive emotions were happy, delighted, and joyful; negative emotions were sad, sorrowful, and depressed; discomfort emotions were tense and nervous. Positive affect (PA), negative affect (NA), and discomfort were separately computed by averaging each of their three corresponding items. A Factor analysis was conducted to explore whether discomfort items are different with the negative emotion items. Both discomfort and negative emotion items were loaded well (i.e., 0.727–0.829) on a single factor (Eigen = 3.942, 65.71% of the variance). But, the loading of tense, anxious, and nervous is higher than sad, sorrowful, and depression. In this study, the relative Cronbach’s *α* values were 0.872 (PA), 0.759 (NA), and 0.758 (discomfort).

We computed each participant’s mixed emotional experience using Schimmack’s (2001) MIN as one of the indexes of mixed emotions. Schimmack (2001) developed a direct measure of co-occurrence, indexing mixed emotions as the smaller one of a given observation’s positive and negative emotion ratings [MIN (positive emotion, negative emotion)]. Higher MIN scores indicate greater mixed emotions, whereas lower scores indicate smaller mixed emotions. We also used the negative acceleration model (NAM; Scott, 1966) as another index of mixed emotions by applying the following formula: ([2 * S] + 1)/(S + L + 2), where S is the smaller, and L is the larger mean affect rating. Higher scores indicate greater mixed emotions (Spencer-Rodgers et al., 2010).

#### Additional Measures

We used one item to measure the extent to which participants experienced conflict, namely, “How conflicted would you feel in this type of event?” Participants rated the item on a 7-point scale from 1 (not at all) to 7 (very strongly).

All the experimental materials were written in English as well as in Chinese and applied to the corresponding native participants. Specifically, the Chinese version was applied after translating and back-translating with the assistance of doctoral candidates in Psychology, and to improve its adherence to Chinese cultural norms, this version underwent some minor adjustments when compared with the original English version.

#### Procedures

Participants were told that this study aimed to assess consumers’ response to potential advertisements. They were instructed to view its appeal just as if they were reading it in a magazine. Randomly, participants were presented with three different advertisements. After reading each advertisement, participants were asked to rate their subjective feelings about the advertisement and respond to the other abovementioned items.

After finishing each emotional measurement, participants were instructed to copy a 3D sketch to avoid the overlapping of the induced emotions. Each emotional measurement took approximately 10 min. After finishing one measurement, the students proceed to the next advertisement. The order of the presentation of the advertisements was counter-balanced.

Subsequently, participants were asked to complete the DSS and a demographic questionnaire. After the questionnaire, participants were debriefed and thanked for their participation.

## Results

### Manipulation Check

The ratings of overall positive and negative emotions were analyzed to check for the general valence of each situation. The results of repeated measurement MANOVA revealed a significant main effect of situation on positive emotion, *F*(1, 315) = 551.277, *p* < 0.01, *η*^2^ = 0.636, and on negative emotion, *F*(1, 315) = 250.207, *p* < 0.01, *η*^2^ = 0.584. Least significance difference analyses indicated that participants in the PPS (*M* = 4.781 ± 0.084) experienced significantly more positive emotions than those in the PUS (*M* = 2.469 ± 0.077), *p* < 0.001; participants in the PUS (*M* = 3.867 ± 0.091) experienced significantly more negative emotion than those in the PPS (*M* = 1.738 ± 0.059), *p* < 0.001. No cultural differences were observed.

For each type of situation, we computed the number of participants that rated conflict greater than 0 (not at all), regardless of the intensity of the emotions. In the PPS, 53.1% of the participants reported perceived contradictions, whereas the rate was 90.3% in the PUS.

### Cultural Differences in Dialectical Thinking

The independent sample *t*-test results showed significant differences between the Chinese and Americans, *t*(315) = 11.422, *p* < 0.01. Specifically, the dialectical thinking of Chinese participants (*M* = 4.275, *SD* = 0.239) was significantly higher than that of American participants (*M* = 3.946, *SD* = 0.273).

One-way ANOVA was conducted to compare the difference between different races on dialectical thinking. The results indicated that there were significant differences between the races, *F*(4, 317) = 33.547, *p* < 0.01. Specifically, dialectical thinking in the Chinese was significantly more prevalent than that in the Anglo- (*M* = 3.932, *SD* = 0.258), African- (*M* = 3.971, *SD* = 0.370), and Hispanic-Americans (*M* = 4.039, *SD* = 0.306); no significant differences were found between the Chinese and Indian-Americans (*M* = 4.072, *SD* = 0.149).

### Cultural Differences in Mixed Emotions

The results of repeated measurement ANOVA showed no significant effects of situation and culture or interaction between them on MIN scores, *p* > 0.05, after controlling for sex, age (Larsen et al., 2007), and multicultural experience (Ritter et al., 2012). The descriptive statistics of the main variables are shown in Table 1.

**Table 1:** Averages and standard deviations of emotions induced by conflict stimuli (*N* = 318).

	Dialectical thinking	PPS					PUS				
		PA	NA	MIN	NAM	Discomfort	PA	NA	MIN	NAM	Discomfort
Americans	3.946 ± 0.273	4.811 ± 1.670	1.733 ± 1.106	1.584 ± 0.901	0.491 ± 1.700	1.516 ± 0.902	2.497 ± 1.504	4.074 ± 1.681	2.051 ± 1.022	0.595 ± 0.173	4.217 ± 1.618
Chinese	4.275 ± 0.239	4.756 ± 1.301	1.748 ± 0.999	1.713 ± 0.897	0.519 ± 0.166	1.883 ± 1.102	2.427 ± 1.209	3.681 ± 1.537	2.019 ± 0.907	0.620 ± 0.163	2.965 ± 1.102

This table presents all the averages and standard deviations of emotions in predominantly pleasant situation and predominantly unpleasant situation. PPS, predominantly pleasant situation; PUS, predominantly unpleasant situation; PA, positive emotions; NA, negative emotions. MIN and NAM represent different calculations of mixed emotions.

A repeated MANOVA indicated no significant main effects of situation and culture or interaction between them on NAM scores, *p* > 0.05, after controlling for sex, age, and multicultural experience.

### Cultural Differences in Discomfort Induced by Conflict Stimuli

**Figure 1:**
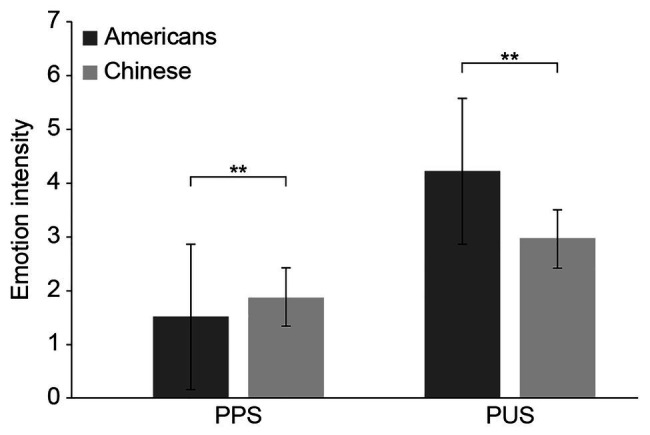
Cultural and situational differences in discomfort induced by conflict stimuli. Condition means of discomfort in predominantly pleasant situation and predominantly unpleasant situation. Error bars indicate 1 standard error of the mean (SE). PPS, predominantly pleasant situation; PUS, predominantly unpleasant situation.^**^p < 0.01.

A repeated measurement ANOVA on discomfort was performed. Figure 1 presents the cultural differences in participants’ discomfort induced by conflict stimuli in two different situations.

After controlling for sex, age, and background, a significant main effect of culture was found, *F*(1, 313) = 128.707, *p* < 0.001, *η*^2^ = 0.291; a main effect of situation, *F*(1, 313) = 8.780, *p* < 0.01, *η*^2^ = 0.027, and culture and situation had significant interactions with discomfort, *F*(1, 313) = 63.996, *p* < 0.001, *η*^2^ = 0.170.

Results of simple effect analysis indicated that: in the PPS, Chinese participants significantly felt more discomfort than Americans, *t*(315) = 3.244, *p* < 0.01, *d* = 0.364; in the PUS, American participants significantly felt more discomfort than Chinese, *t*(315) = −7.087, *p* < 0.001, *d* = 0.796.

### Mediation of Dialectical Thinking in the Relationship Between Culture and Mixed Emotional Experience

The correlation of all variables mentioned before is shown in Table 2. We conducted mediation analyses on the two different situations separately (PPS and PUS).

**Table 2:** Correlations between dialectical thinking and emotional experience induced by conflict stimuli (*N* = 318).

	1	2	3	4	5	6	7	8	9	10	11	12
1. Culture	1											
2. Dialectical thinking	−0.541^**^	1										
3. PA1	0.018	0.027	1									
4. NA1	−0.007	0.087	−0.208^**^	1								
5. MIN1	−0.072	0.118^*^	−0.044	0.876^**^	1							
6. NAM1	−0.086	0.108	−0.543	0.726^**^	0.825^**^	1						
7. Discomfort1	−0.180^***^	0.213^**^	0.010	0.613^**^	0.676^**^	0.516^**^	1					
8. PA2	0.026	0.053	0.241^**^	0.068	0.115^*^	−0.030	0.188^**^	1				
9. NA2	0.122*	−0.018	0.214^**^	0.214^**^	0.202^**^	0.038	0.130^*^	−0.288^**^	1			
10. MIN2	0.017	0.026	0.244^**^	0.237^**^	0.287^**^	0.097	0.348^**^	0.646^**^	0.157^**^	1		
11. NAM2	−0.074	0.013	0.035	0.114^*^	0.159^**^	0.109	0.248^**^	0.420^**^	−0.355^**^	0.773^**^	1	
12. Discomfort2	0.371^**^	−0.126^*^	0.150^**^	0.185^**^	0.195^**^	0.087	0.158^**^	−0.046	0.647^**^	0.210^**^	−0.152^**^	1

PA1 represents the positive emotions in a predominantly pleasant situation. NA1 represents the negative emotions in a predominantly pleasant situation. MIN1 and NAM1 represent the mixed emotional experience in a predominantly pleasant situation. Discomfort1 represents the tense and nervous induced in a predominantly pleasant situation. PA2 represents the positive emotions in a predominantly unpleasant situation. NA2 represents the negative emotions in a predominantly unpleasant situation. MIN2 and NAM2 represent the mixed emotional experience in a predominantly unpleasant situation. Discomfort2 represents the tense and nervous induced in predominantly unpleasant situation.

* *p* < 0.05

** *p* < 0.01

*** *p* < 0.001.

**Figure 2:**
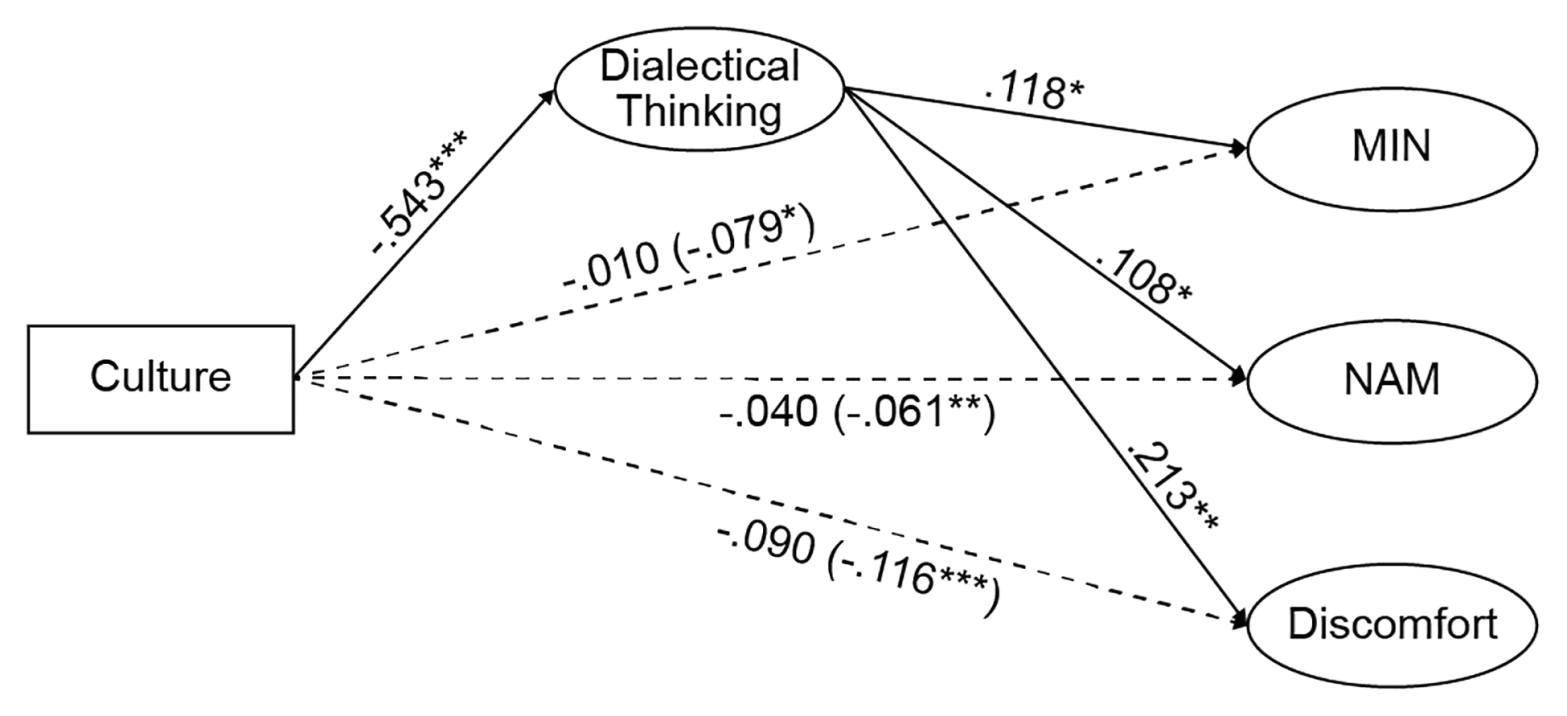
Mediation scheme of dialectical thinking in the association between culture and mixed emotional experience (MIN, NAM, and discomfort) in the predominantly positive situation. Illustrated values depict the standardized path parameters of mediation model in predominantly pleasant situations. Chinese participants were coded as 0; American participants were coded as 1; culture served as the independent variable; dialectical thinking as the mediation variable; MIN, NAM, and discomfort in predominantly pleasant situation as the outcome variables; sex and age were control variables. Values in brackets depict the indirect effects of culture on three different predictors. ^*^p < 0.05; ^**^p < 0.01; ^***^p < 0.001.

In the PPS, results indicated that culture significantly negatively predicted dialectical thinking, *β* = −0.543, *p* < 0.001. When culture and dialectical thinking both entered the model, dialectical thinking significantly positively predicted MIN, *β* = 0.188, *p* < 0.05, and discomfort, *β* = 0.213, *p* < 0.01. The direct effect of culture was not significant on MIN, NAM, and discomfort, *β*_MIN_ = −0.01, *β*_NAM_ = −0.04, *β*_DIS_ = −0.09, *p* > 0.05. The Bootstrap confidence intervals of the indirect effect of culture on MIN were [−0.121, −0.009], indicating that dialectical thinking played a significant indirect effect on the relationship between culture and MIN, *β* = −0.079, *p* < 0.001. The Bootstrap confidence intervals of the indirect effect of culture on NAM were [−0.117, −0.001], indicating that culture had a significant indirect effect on NAM, *β* = −0.061, *p* < 0.001. The Bootstrap confidence intervals of the indirect effect of culture on discomfort were [−0.173, −0.059], indicating that culture had a significant indirect effect on discomfort, *β* = −0.116, *p* < 0.001. Figure 2 shows the standardized values of our mediation model, and the model fit indexes are shown in Table 3.

**Table 3:** Model fit indexes of the mediation analysis in the predominantly positive situation.

	CMIN/DF	NFI	RFI	IFI	TLI	CFI	RMSEA
Model	1.34	0.994	0.981	0.999	0.995	0.998	0.033

However, in the PUS when culture and dialectical thinking both entered the model, the effects of dialectical thinking on MIN, NAM, or discomfort were not significant, *β* = −0.031, *p* > 0.05. Similarly, the direct effects of culture on MIN, NAM, or discomfort were not significant.

## Discussion

The present study investigated the cultural differences in people’s mixed emotional experiences, including its byproducts (discomfort induced by conflict stimuli in different situations), and explain how differing thinking styles affect these experiences. The initial findings show no cultural differences in mixed emotions between the two groups, but cultural differences in discomfort. Dialectical thinking mediated these cultural differences in mixed emotional experience and discomfort. These results support our hypotheses. The present study expands on previous studies by exploring the role of dialectical thinking in the cultural differences in mixed emotion and its byproduct (discomfort).

### Cultural Differences in Mixed Emotional Experiences in Different Situations

We analyzed the interaction between culture and advertisement messaging on different indexes of mixed emotions, MIN and NAM, as well as discomfort. No significant cultural differences were found in either MIN or NAM. Thus, both indexes of mixed emotions provided consistent evidence that Americans and Chinese experienced the same mixed emotions in the PPS and the PUS, and that Chinese felt more mixed emotions than Americans only in the PUS. The findings indicated that both Americans and Chinese have the ability to perceive and experience the mixed emotional stimuli; hence, when confronted with conflict, they both experience conflicting emotions. These findings support our hypothesis (H1) and coincide with those of Leu et al. (2010) and provide a valuable supplement to Spencer-Rodgers et al. (2010) work. Further, we also compared the cultural differences in different indexes of mixed emotions and found consistent evidence (Spencer-Rodgers et al., 2010).

However, results also showed that the same stimuli made Chinese and American participants experience different degrees of discomfort. First, the discomfort rose as the intensity of mixed emotions increased in both situations; however, the discomfort increased more in the PUS than in the PPS. Second, in the PPS, Chinese reported significantly more discomfort than Americans; in the PUS, Americans reported significantly more discomfort than Chinese.

This pattern of results has several important implications. First, the findings show that both the American and Chinese participants experienced comparable mixed emotions in response to positive and negative situations. Previous research on culture and mixed emotions has tended to neglect situational factors; we hope our situational account may explain some previous inconsistent findings on culture and mixed emotions (Hui et al., 2009; Miyamoto et al., 2010). Second, we explored the association between culture and discomfort that accompanies mixed emotions. Though previous studies claim that mixed emotions can make one tense and nervous, little attention has been paid to the cultural differences of discomfort when individuals were confronted with conflicting stimuli. To our knowledge, this study is the first attempt to explore the cultural differences in discomfort that accompany mixed emotions to deepen extant understandings of the experience of mixed emotions.

### Mediation of Dialectical Thinking in Cultural Differences in Mixed Emotional Experiences

Why do American and Chinese people experience comparable mixed emotions, but different levels of discomfort in both positive and negative situations? Peng and Nisbett (1999) have suggested that a dialectical style of thinking may explain the cultural differences in contradiction processing. One plausible alternative mechanism is that because the Chinese tend to engage in dialecticism they therefore habitually process both positive and negative information with more ease.

The results of mediation analyses indicated that dialectical thinking does indeed play a complete mediating role in the impact of cultural differences in MIN, NAM, as well as in discomfort in the PPS. We believe that the PPS findings may be related to features of dialecticism, namely, the expectation for change and tolerance of contradictions (Peng and Nisbett, 1999). Dialecticism also dictates that the valence of an event is likely to change with time and perspective (Ji et al., 2001). This means that, when confronted with positive events, the Chinese tend to think that such happiness might endure for a short time, so they start looking for potential negative implications, that is, they start planning ahead for a future rainy day. Contrastingly, Americans tend to maximize the happy moment they are living because they tend to think they are currently experiencing it owing to a deeply rooted principle in the American culture: “Happiness is a basic right that everyone can pursue”; this principle detracts from paying too much attention to the fact that such positivity will not last forever.

Nevertheless, in the PUS, the mediating effect of dialectical thinking on cultural differences in mixed emotional experience was not observed. This finding denotes that dialectical thinking can interpret the cultural differences in mixed emotional experience in PPS, which is in accordance with the previous research that dialectical thinkers and non-dialectical thinkers differ in their level of mixed emotions only in positive events, but not in negative events (Hui et al., 2009). This may due to different mechanisms. Dialectical thinkers may be driven by their balanced event appraisal style to experience mixed emotions, whereas non-dialectical thinkers may be motivated by their self-affirmation to generate positive implication to cope with negative events (Larsen et al., 2003).

These results shed light on a thinking style account for why some individuals experience mixed emotions more discomfort. Previous studies explore the association of dialecticism and mixed emotional experience, which is correlational, and cannot draw causal inferences (Hui et al., 2009). In this study, we introduced the mixed emotional experience and provided empirical support for the associations. This study addressed how an ideological thinking style can explain the cultural differences in mixed emotions and provided us a further insight into how people process and experience the ambivalence in different cultures. When studying mixed emotions in a global context, the cultural factors and the thinking style factors should be taken into account.

## Conclusion

This study suggests that the impact that cultural differences have on people’s mixed emotional experiences depends on the emotional components of mixed emotional situations. Further, it suggests that the American and Chinese both experience mixture of emotions in different valence situations, but their discomfort toward the conflicting stimuli is different. Additional dialectical thinking may be a mechanism behind the influence of cultural differences in people’s mixed emotional experiences. Together, the findings contribute toward future theorizing on social cognition and emotion.

## Data Availability Statement

The datasets generated for this study can be found in the https://mfr.de-1.osf.io/render?url=https://osf.io/zrt3k/?direct%26mode=render%26action=download%26mode=render.

## Ethics Statement

The studies involving human participants were reviewed and approved by Human Research Ethics Committees of both Capital Normal University and University of California Berkeley. The patients/participants provided their written informed consent to participate in this study.

## Author Contributions

WZ, AY, and DL contributed equally to this work. WZ and KP served as the corresponding authors for the manuscript. PF and KP contributed significantly to the theory development, with KP contributing to research resources. DL contributed to the initial research design and data collection. WZ contributed to additional data collection, data analysis, as well as drafting and revising of the manuscript. AY contributed to the additional data collection, as well as the drafting and revising of the manuscript. All authors contributed to the article and approved the submitted version.

### Conflict of Interest

The authors declare that the research was conducted in the absence of any commercial or financial relationships that could be construed as a potential conflict of interest.
